# Supplementary figures and images for: Comparative Study of SARS-CoV-2, SARS-CoV-1, MERS-CoV, HCoV-229E and Influenza Host Gene Expression in Asthma: Importance of Sex, Disease Severity, and Epithelial Heterogeneity
Source: Viruses. 2021 Jun 5;13(6):1081. doi: 10.3390/v13061081 (PMC8226441; doi:10.3390/v13061081)

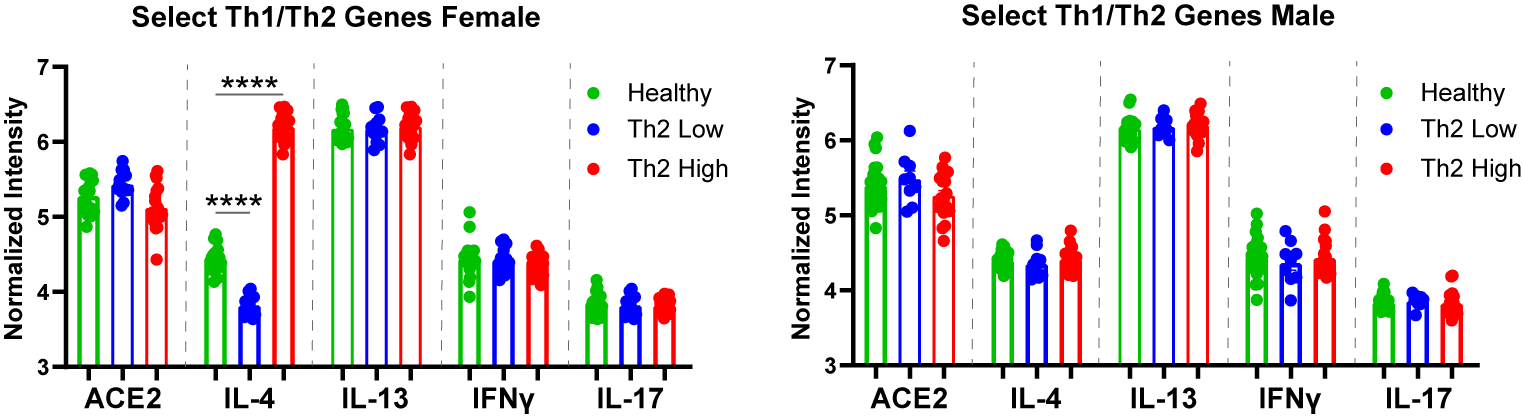

Supplement: Supplementary file 1 [file viruses-13-01081-s001.zip › viruses-1222925-supplementary.tif]
